# HIF repressors under chronic hypoxia

**DOI:** 10.18632/aging.100922

**Published:** 2016-03-05

**Authors:** Weibo Luo, Yingfei Wang

**Affiliations:** Departments of Pathology and Pharmacology, UT Southwestern Medical Center, Dallas, TX 75390, USA

**Keywords:** chronic hypoxia, hypoxia-inducible factor, peroxiredoxin, gene regulation, transcription corepressor

Decreased ability in oxygen delivery and diffusion of the blood vessels and increased oxygen consumption in the rapidly proliferating cancer cells both contribute to reduced oxygen availability in the solid tumors, a major signature of the tumor microenvironment [1]. The oxygen concentration is dynamically changed within the tumor, and thus tumor cells are frequently subjected to acute or chronic hypoxia. Tumor cells exposed to chronic hypoxia are highly malignant and resistant to radiation therapy and chemotherapy [1]. Hypoxia-inducible factor 1 (HIF-1) and HIF-2 are two master regulators in response to low oxygen and mediate tumor progression by regulating the transcription of target genes [2]. HIF-mediated cellular processes are distinct under conditions of acute and chronic hypoxia, which may have great impact on therapeutic consequences. In this editorial, we will discuss the negative HIF regulators that differentially control HIF transcriptional activity under conditions of acute and chronic hypoxia.

HIF is a heterodimeric transcription factor, consisting of an oxygen-regulated α subunit and a constitutively expressed β subunit. The regulation of HIF-α protein levels is a critical determinant of HIF-mediated gene transcription. Our previous study showed that chronic hypoxia causes the selective decay of HIF-1α at least in part by HSP70- and CHIP-dependent ubiquitination and proteasomal degradation [3]. The levels of HIF-1α mRNA are also suppressed by repressor element 1-silencing transcription factor (REST) [4], miR-155 [5], or tristetraprolin [6] under conditions of chronic hypoxia. Apart from decreased mRNA and protein stability, we recently found that HIF transcriptional activity is directly inhibited by peroxiredoxin 2 (PRDX2) and PRDX4 under conditions of chronic hypoxia [7]. PRDX2 mRNA and protein levels are elevated in HeLa cells exposed to chronic hypoxia in a HIF-1- and HIF-2-dependent manner [7]. Chronic hypoxia also increases the nuclear translocation of PRDX2 and PRDX4 in HeLa cells [7]. The nuclear PRDX2 or PRDX4 physically binds to the inhibitory domain of HIF-1α and HIF-2α, and directly inhibits the transcriptional activity of HIF-1 and HIF-2 in multiple cell lines, including human cervical carcinoma HeLa, human embryonic kidney HEK293T, and mouse embryo fibroblasts [7]. The antioxidant activity of PRDX2 and PRDX4 are dispensable for HIF suppression [7].

Moreover, we further found that double knockdown of PRDX2 and PRDX4 selectively increases expression of a subset of HIF target genes, including *SLC2A3*, *GPI*, *PDK3*, *HGF*, in HeLa cells exposed to chronic hypoxia (1% O_2_ for 72 hr) [7]. However, PRDX2 and PRDX4 fail to affect the mRNA levels of these genes under conditions of acute hypoxia (1% O_2_ for 24 hr) [7]. Mechanistically, PRDX2 and PRDX4 selectively impair HIF-1 and HIF-2 binding to the hypoxia response element of a subset of HIF target genes, thereby inhibiting gene expression in cells under conditions of chronic hypoxia [7]. Future studies are required to determine whether PRDX2 and PRDX4 are involved in recruitment of the HIF corepressor complex to the HIF target genes. Nevertheless, our findings indicate that PRDX2 and PRDX4 represent a novel feedback mechanism for inhibition of HIF transcriptional activity under conditions of chronic hypoxia.

HIFs have been the attractive targets for cancer therapy. Identification of the specific HIF regulators under conditions of acute and chronic hypoxia may yield the new therapeutic strategies. Indeed, high levels of PRDX2 and PRDX4 are significantly correlated with increased survival of patients with bladder cancer (our unpublished data), suggesting that PRDX2 and PRDX4 may be the new therapeutic targets for treatment of cancer.
